# Lignin-Mediated Dual Conductive Hydrogels with High Conductivity, Antibacterial Activity and Biocompatibility for Chronic Wound Repair

**DOI:** 10.3390/gels11040283

**Published:** 2025-04-11

**Authors:** Jianhong Lin, Mengyao Chen, Wei Zhao, Shengyu Zhang, Jialin Liu, Yang Zhou, Lei Jiang, Jiantao Zhang

**Affiliations:** 1 Laboratory of Advanced Theranostic Materials and Technology, Ningbo Institute of Materials Technology and Engineering, Chinese Academy of Sciences, Ningbo 315201, China; 2 University of Chinese Academy of Sciences, Beijing 100049, China; 3 School of Materials Science and Chemical Engineering, Ningbo University, Ningbo 315211, China; 4 Cixi Biomedical Research Institute, Wenzhou Medical University, Cixi 315302, China

**Keywords:** lignin sulfonate, polypyrrole, silver nanoparticles, conductive hydrogels, chronic wound

## Abstract

In recent years, conductive polymer hydrogels based on polypyrrole (PPy) combined with electrical stimulation (ES) have emerged as a promising approach for chronic wound repair. However, in practical applications, PPy often exhibits limitations such as poor water dispersion, weak inherent conductivity and a lack of biological functionality. To address these challenges, this study proposes an innovative design of a conductive hydrogel that employs a natural biopolymer, lignin sulfonate (Lgs), as both a dispersant and dopant for PPy, while incorporating silver nanoparticles (Ag NPs) to confer the hydrogel antibacterial properties. The results showed that the water dispersion of PPy was significantly improved, and the conductivity of the hydrogel was as high as 2.82 ± 0.04 mS/cm through the double conduction mechanism of PPy and Ag NPs. The hydrogel exhibited antibacterial activity against Escherichia coli (*E. coli*) and Staphylococcus aureus (*S. aureus*), and the antibacterial rate could exceed 90%. In vitro tests demonstrated that the hydrogel exhibited good biocompatibility, adhesion ability (7.97 ± 0.56 kPa) and hemostatic ability. Furthermore, in vivo animal experiments showed that the hydrogel combined with ES achieved 93.71 ± 2.46% wound closure within 14 days, which can significantly accelerate wound healing, promote collagen deposition and epithelial tissue regeneration. These findings demonstrate that the developed hydrogel can serve as an effective platform for ES-assisted chronic wound repair.

## 1. Introduction

Chronic wounds, such as pressure ulcers, diabetic ulcers, venous leg ulcers, etc., have significant morbidity and mortality among patients and have become a serious problem to the healthcare system [1,2]. Wound closure typically goes through four consecutive steps, hemostasis, inflammation, proliferation and remodeling, which are regulated by multiple and cascaded physiological processes [3,4]. This well-organized process is disrupted or delayed in chronic wounds, leading to the ineffectiveness of traditional wound management in promoting the wound healing process [5,6]. In recent years, electrical stimulation (ES) has emerged as a promising, non-invasive and controllable therapeutic approach for promoting wound healing, demonstrating significant advancements. By modulating proliferation, migration, differentiation and gene expression, ES can accelerate wound repair [7]. In the treatment of chronic wounds, ES has shown particularly notable efficacy by promoting angiogenesis, alleviating inflammation and accelerating epithelial regeneration, ultimately enhancing the healing process [8,9,10]. However, existing ES-based therapies face several challenges, especially concerning the selection and optimization of electrode materials.

Currently, electrodes used in ES therapy can be broadly categorized into rigid and flexible types. Rigid electrodes typically possess excellent electrical conductivity but often fail to adapt to the contours of skin or wound surfaces, leading to uneven stimulation and potential skin damage [11,12,13]. Furthermore, their mechanical rigidity and poor adaptability make them uncomfortable for extended wear [14]. In contrast, flexible electrodes, with their better conformability and comfort, can provide more uniform electrical stimulation, making them particularly suitable for soft tissue applications like skin repair [15,16,17]. However, flexible electrodes often suffer from lower conductivity, reduced stability and compromised performance in wet environments, limiting their widespread application in wound healing [18].

To address these issues, conductive hydrogels have attracted considerable attention as promising flexible electrode materials due to their unique advantages [19,20]. These hydrogels not only conform seamlessly to wound surfaces but also maintain a moist environment, which aligns with the theory of moist wound healing [21]. Moreover, their flexibility and biocompatibility ensure minimal irritation to the surrounding tissues [22,23]. The inherent water content of hydrogels also facilitates ion conduction, making them highly suitable for ES-based wound therapy. Despite these benefits, current conductive hydrogels face challenges, particularly regarding inadequate conductivity and limited biological functionalities, such as antimicrobial properties and bioactivity, which are crucial for preventing infections and ensuring timely healing [24,25].

Various strategies have been developed to fabricate conductive hydrogels, including the incorporation of conductive polymers, ionic conductive components and inorganic conductive materials [26,27]. Among them, polypyrrole (PPy) has excellent biocompatibility, chemical stability and electrical conductivity, which makes it an ideal component for conductive hydrogels in biomedical applications [28]. Despite their advantages, PPy-based conductive hydrogels face several limitations hindering their broader applications. For example, Lin et al. [29] prepared a conductive hydrogel by in situ polymerization of PPy in a collagen-based hydrogel. However, in this way, the hydrophobic structure of PPy would cause its uneven dispersion within the hydrogel system, resulting in unstable electrical properties. Zhang et al. [30] grafted PPy onto double-bond modified chitosan molecules by free radical grafting polymerization. Although this method improved the dispersion of PPy in the hydrogel system, PPy was not protonated or charge transfer doped, and was in the eigenstate with limited conductive properties. Distler et al. [31]. combined (Polystyrene sulfonic acid) PSS and PPy to improve the water dispersion and electrical conductivity of PPy; however, the high content of PSS would lead to an acidic physiological environment, resulting in wound irritation. In addition, the currently reported conductive hydrogels based on PPy still lack certain biological activities, such as limited antibacterial ability [32]. Therefore, enhancing the water dispersion of PPy, optimizing its electrical properties in the eigenstate and making it have good biological activity remain key challenges in the field of conductive hydrogels.

To address these limitations, this study proposed an innovative conductive hydrogel design. First, lignin sulfonate (Lgs), a natural biopolymer, was incorporated into the conductive hydrogel system. The -OH and -OSO_3_H groups on Lgs molecules electrostatically interact with the -NH groups of polypyrrole (PPy), enabling PPy to align along Lgs chains and significantly enhancing PPy aqueous solubility. Furthermore, the -OSO_3_H groups serve as anionic dopants, which modify the electronic structure of PPy and facilitate electron transport, thereby substantially improving the conductivity of PPy [33]. Moreover, the inherent reducing capability of Lgs facilitates the in situ reduction of silver ions into silver nanoparticles (Ag NPs), which further enhances the conductivity and antibacterial properties of hydrogels. This dual conductive mechanism—electronic conductivity from PPy and Ag NPs—ensures superior performance under electrical stimulation. Additionally, the antimicrobial effect of Ag NPs helps prevent wound infections, thereby accelerating chronic wound healing. Finally, polyvinyl alcohol (PVA), which is biocompatible, safe and non-toxic and widely used in biomedical medicine, was selected as the hydrogel matrix material [34]. By integrating high conductivity, antimicrobial properties and excellent biocompatibility, this study provides a novel, efficient material platform for ES-based therapies, promoting the clinical adoption of this technology and improving therapeutic outcomes for patients with chronic wounds.

## 2. Results and Discussion

### 2.1. Material Design and Characterizations

**Figure 1:**
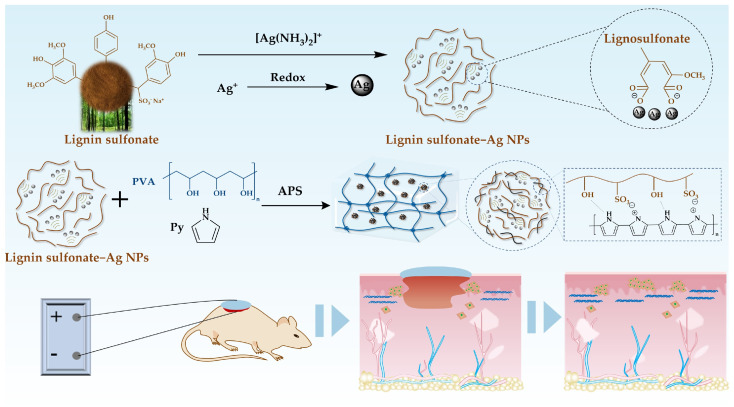
Schematic diagram illustrating the formation and application of PLAP hydrogel as a multifunctional dressing for chronic skin wound healing.

Lgs and Ag NPs were incorporated into the hydrogel system to address the insufficient conductive ability and limited antibacterial properties of PPy. Lgs serves not only as a dopant for PPy, enhancing its water dispersion, but also facilitates the reduction of silver ions to generate Ag NPs, thereby improving both the conductivity and antibacterial properties of the hydrogels. The specific formation strategies of PLAP hydrogels are illustrated in Figure 1. During the synthesis of Lgs-Ag NPs, Lgs exhibits a variety of properties, including reduction ability and dispersant ability, due to its complex structure and diverse functional groups (such as alcohol hydroxyl, phenol hydroxyl and sulfonic acid groups) [35]. When [Ag (NH_3_)_2_]^+^ was introduced, the phenol hydroxyl group on Lgs adsorbed Ag^+^ and reduced Ag^+^ to metallic silver atoms. These silver atoms would aggregate in an orderly way in the three-dimensional network of Lgs, regulated by the charged groups in the Lgs, forming Ag NPs with uniform particle size and narrow distribution [36]. In the reduction process of Ag^+^, the corresponding phenolic hydroxyl in Lgs was oxidized into an unstable C=O group and further converted into a carboxyl group, which was also conducive to controlling the synthesis process of Ag NPs by electrostatic action [37]. Additionally, the hydrophilic and hydrophobic groups present in Lgs effectively disperse and stabilize Ag NPs, preventing aggregation [38].

**Figure 2:**
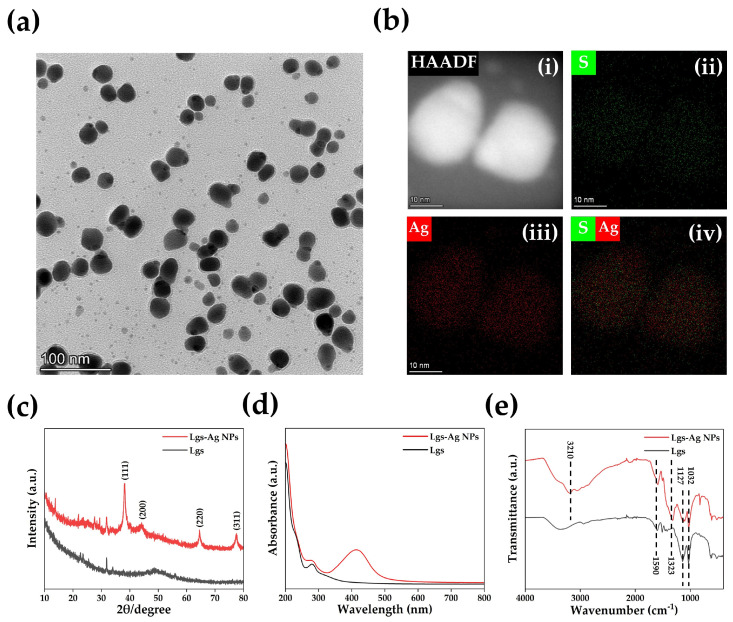
Characterization of Lgs-Ag NPs. (**a**) TEM morphologies of Lgs-Ag NPs. ((**b**) (**i**) HAADF-HRTEM image of Lgs-Ag NPs. (**ii**) S high-resolution elemental distribution maps of Lgs-Ag NPs. (**iii**) Ag high-resolution elemental distribution maps of Lgs-Ag NPs. (**iv**) S, Ag high-resolution elemental distribution maps of Lgs-Ag NPs). (**c**) UV−Vis absorption spectroscopy analysis of Lgs and Lgs-Ag NPs. (**d**) XRD patterns of samples of Lgs and Lgs-Ag NPs. (**e**) FTIR spectra of Lgs and Lgs-Ag NPs.

Subsequently, pyrrole (Py), PVA and APS were successively added into the Lgs-Ag NPs solution using a one-pot method. The concentration of PVA was determined by previous studies (Figure S1). The PLAP hydrogels were prepared through multiple freeze–thaw cycles. During the polymerization process, Py was affected by the phenol hydroxyl, methoxy and sulfonic acid groups of Lgs. These functional groups ensure uniform dispersion of PPy in the hydrogel system through intermolecular interactions with the protonated nitrogen groups of PPy [39]. Additionally, sulfonic acid groups, acting as dopants, are attracted to free radicals formed during the oxidation of Py through local resonance and combine into polarons, which changes the energy state structure of conductive polymers and promotes the movement of charge carriers from the highest occupied molecular orbital (HOMO) to the lowest unoccupied molecular orbital (LUMO) [33]. This process enhances electron mobility along the polymer chain, thereby improving the conductive properties of materials. Figure 2 demonstrates the characterization of Lgs-Ag NPs. As shown in Figure 2a, the Lgs-Ag NPs were evenly dispersed in the visual field, with an average particle size of 27.07 ± 6.57 nm (Figure S2). Energy dispersive X-ray spectroscopy (EDS) was employed to present the internal structure and elemental distribution of Lgs-Ag NPs (Figure 2b). The high-angle annular dark field (HAADF) image reveals the Lgs-Ag NPs (Figure 2b(i)), and the elemental mapping indicated that Ag and S elements were evenly distributed within the Lgs-Ag NPs, confirming the successful combination of Lgs and Ag (Figure 2b(ii–iv)). The optical properties of Lgs-Ag NPs were characterized by UV–Vis absorption spectroscopy (Figure 2c). Lgs-Ag NPs solution exhibited a broad absorbance band centered at approximately 415 nm, which is attributed to the surface plasmon resonance of Ag NPs [40]. In contrast, Lgs solution only showed a characteristic absorption peak corresponding to free phenol groups at 280 nm [41]. The XRD pattern of Lgs-Ag NPs is shown in Figure 2d. The XRD pattern of the Lgs-Ag NPs sample exhibited characteristic peaks at 2θ = 38.20°, 44.17°, 64.53° and 77.51°, which correspond to the (111), (200), (220) and (311) crystal planes of silver crystals, respectively. These results are in agreement with previous reports by Ahmad et al. [42]. As shown in Figure 2e, the chemical structures of Lgs-Ag NPs and Lgs were further characterized by Fourier-transform infrared (FTIR) spectroscopy. The characteristic peak of the phenolic hydroxyl group was observed at 3210 cm^−1^ in Lgs-Ag NPs, while the skeletal vibration of the aromatic ring was detected at 1600 cm^−1^ [43]. The peaks at 1032 cm^−1^ and 1137 cm^−1^ were assigned to the S-O stretching vibration and S=O symmetric stretching vibration, respectively [44]. Additionally, a new peak at 1323 cm^−1^ was identified as the symmetric stretching vibration of -COO^−^, indicating Ag^+^ induced oxidation of Lgs. These results demonstrated that Lgs-Ag NPs were successfully synthesized.

### 2.2. Properties of the Hydrogels

**Figure 3:**
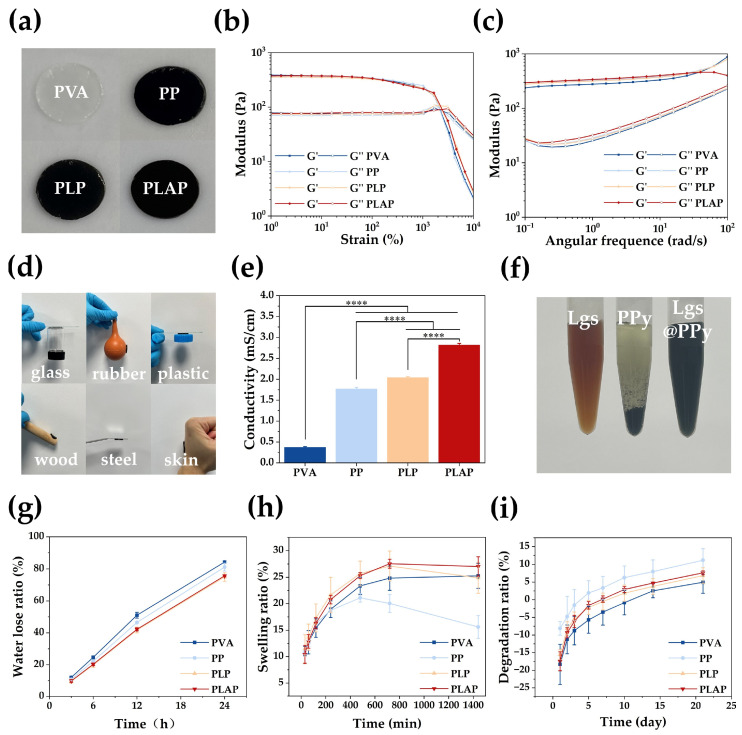
Rheological, adhesive and conductive properties of hydrogels. (**a**) Photographs of the PVA, PP, PLP and PLAP hydrogels. Storage modulus (G′) and loss modulus (G″) of PVA-based hydrogels under (**b**) oscillatory strain amplitude sweep and (**c**) frequency sweep. (**d**) Photographs of PLAP hydrogels adhered to various substrates such as glass, rubber, plastic, wood, steel and skin. (**e**) Electrical conductivity of PVA-based hydrogels. (**f**) Photographs of Lgs, PPy and Lgs@ PPy dispersed in water. (**g**) Water retention properties of hydrogels. (**h**) Swelling properties of hydrogels. (**i**) Degradation properties of hydrogels. A *p*-value of ≤ 0.05 was considered significant (**** *p* < 0.0001).

Multi-functional PVA-based hydrogels with conductivity, antibacterial activity and biocompatibility were prepared through physical cross-linking of molecular chains formed by freezing and thawing of PVA. The PVA hydrogels were translucent white, whereas PP, PLP and PLAP hydrogels were black due to the incorporation of PPy (Figure 3a). As shown in Figure S3a, all PVA-based hydrogels exhibited three-dimensional porous structures. Notably, the three-dimensional porous structure of the hydrogels promotes cell proliferation, migration and nutrient exchange, thereby facilitating rapid wound healing. In addition, the porosity of each hydrogel was further tested via an absolute ethanol method. As shown in Figure S3b, all hydrogels have about 30% porosity.

Furthermore, the viscoelasticity and stability of each hydrogel were evaluated by rheological characterization. First, the linear viscoelastic region was determined by strain amplitude scanning measurements. As shown in Figure 3b, in the linear viscoelastic domain, all hydrogels showed storage modulus (G′) > loss modulus (G″). Their mechanical properties were also characterized through viscoelasticity measurements. A wider linear viscoelastic region corresponds to greater strain resistance in the material. When the strain range was 1–2100%, all hydrogels exhibited G′ > G″. This indicates that the hydrogels have good strain resistance, preserving their structural integrity under mechanical stress, thus effectively protecting the wound and reducing secondary tissue damage caused by external pressure. In the angular frequency range of 0.1~100 rad/s, all of the hydrogels showed G′ > G″ (Figure 3c), indicating that they had solid-like properties and could resist the shear force encountered during the wound healing process, thereby effectively protecting the wound from external damage.

Hydrogels with adhesive properties can be effectively attached to the wound, preventing water loss and protecting against external stimulation and damage. As shown in Figure 3d, PLAP hydrogels could adhere firmly to the surfaces of glass, rubber, plastic, wood, steel and skin tissue without detachment. In addition, the adhesive strength of the PLAP hydrogel was determined to be 7.97 ± 0.56 kPa through lap shear testing (Figure S4).

The conductivity of each hydrogel was characterized via a four-probe method. As shown in Figure 3e, the conductivities of the PVA, PP, PLP and PLAP hydrogels progressively increased. Due to the absence of any conductive component, PVA hydrogels had the lowest conductivity of 0.38 ± 0.02 mS/cm. The addition of PPy increased the conductivity of PP to 1.77 ± 0.04 mS/cm. Furthermore, the introduction of Lgs as a dopant altered the energy state structure of PPy and enhanced charge mobility. Simultaneously, molecular interactions between the phenol hydroxyl, methoxy and sulfonic acid groups of Lgs and the protonated nitrogen groups of PPy promoted the uniform distribution of PPy along the Lgs, improving its water dispersion and preventing aggregation and sedimentation (Figure 3f and Figure S5). The hydrogel conductive network was optimized and the conductivity was improved (2.04 ± 0.02 mS/cm). To further enhance the conductivity of the hydrogels, Ag NPs were incorporated into the hydrogel system, combining organic and inorganic electronic conductors to achieve a maximum conductivity of 2.82 ± 0.04 mS/cm for the PLAP hydrogels. In addition, in a series circuit, it could be used as a good conductor to make LED bulbs brighter (Figure S6). Skin is an electrically sensitive tissue with conductivity values between 0.1 and 2.6 mS/cm [45]. Skin tissue lesions impede signal transduction and prevent cellular behavior. Therefore, hydrogels with excellent electrical conductivity can improve signal transduction at wound sites, induce cell behavior, promote cell proliferation, migration and other processes, and accelerate wound healing.

The moisture retention capacity of the hydrogels was quantitatively assessed by measuring their water loss ratio over time. As shown in Figure 3g, all hydrogel formulations exhibited progressively increasing water loss ratios during the 24 h evaluation period. Notably, PLP and PLAP hydrogels demonstrated significantly lower water loss rates compared to PVA and PP hydrogels. This enhanced water retention capability can be attributed to the polyhydroxyl structure of Lgs, which effectively reduces water evaporation and improves the moisture retention properties of the hydrogels. After that, the swelling performance of the hydrogels was evaluated (Figure 3h). PP hydrogels exhibited the lowest swelling ratio, attributable to the hydrophobic nature of PPy, which limited water absorption. In contrast, PLP and PLAP hydrogels demonstrated superior swelling performance, reaching 27.11 ± 2.80% and 27.56 ± 0.85% within 12 h, respectively, due to the incorporation of hydrophilic lignosulfonate Lgs. These results suggest that PLP and PLAP hydrogels can effectively absorb wound exudate while maintaining structural stability, preventing excessive swelling-induced wound compression.

The degradation behavior of hydrogels is an important factor for wound dressing applications. As shown in Figure 3i, all hydrogel groups exhibited an initial mass increase due to ongoing swelling. Subsequently, gradual degradation occurred, with cumulative mass loss remaining below 10% over 21 days. The degradation rates followed the order PP > PLAP > PLP > PVA. These results indicate that these hydrogels maintain structural integrity for extended periods, making them suitable for long-term wound healing applications.

### 2.3. Biocompatibility of Hydrogels

**Figure 4:**
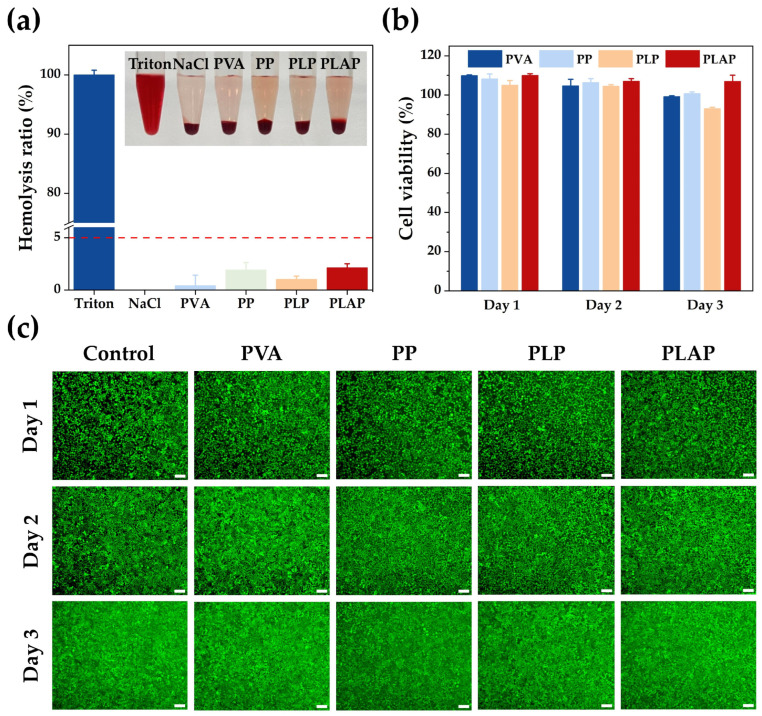
In vitro hemocompatibility and cytocompatibility. (**a**) Hemolysis analyses of PVA-based hydrogels (n = 3). (**b**) Proliferation of L929 cells cultured in hydrogel extract for 1, 2 and 3 days. (**c**) Fluorescence images of L929 cells cultured in hydrogel extract for 1, 2 and 3 days (Scale bar = 100 μm, n = 3).

As a wound repair material, hydrogel dressings should have biocompatibility. In this study, the biocompatibility of hydrogels was assessed at both cellular and blood levels (Figure 4).

Initially, mouse fibroblast (L929) cells were used to assess the biocompatibility of the hydrogels. The hydrogel extract was co-cultured with L929 cells, and the cell viability was detected with a CCK-8 assay. The reduction of WST-8 to water-soluble formazan by cellular dehydrogenases was measured at 450 nm, which indirectly reflects cell viability through absorbance. As shown in Figure 4b, after 24 h co-culture with cells, the cell viability of all groups reached 100%, and when the culture time was extended to 48 or 72 h, the cell viability remained above 80%, in accordance with international biomaterial standards (ISO 10993-1: 2018 [46]. Furthermore, cell viability was qualitatively evaluated using Calcein-AM (Beyotime, Shanghai, China)staining. In this assay, viable cells containing active esterases effectively converted Calcein-AM into fluorescent Calcein, while dead cells, lacking enzymatic activity, could not complete this conversion. The staining results are presented in Figure 4c. After treatment with hydrogel, cells in each group displayed extensive green fluorescence. Cell morphology showed no significant difference compared to the control group, indicating that the hydrogels exhibited excellent biocompatibility, consistent with the CCK-8 results.

According to the international standard ISO 10993-4: 2018 [46], materials used in the biomedical field need to have a hemolysis rate of less than 5%. The hemolysis rate test of each hydrogel was performed with fresh rabbit blood. 1% Triton X-100 was considered a positive control, inducing complete hemolysis (bright red supernatant), while a 0.9% NaCl solution was considered a negative control, resulting in complete non-hemolysis (colorless supernatant). As shown in Figure 4a, the supernatant of each group after hydrogel treatment was nearly colorless. Among these, the hemolysis rates for PP and PLAP hydrogels were 1.92 ± 0.71% and 2.1 ± 0.38%, respectively, which were similar to the hemolysis rates of dextrin–gelatin hydrogel (1.83 ± 0.07%), alginate–gelatin hydrogel (1.71 ± 0.02%), chitosan–starch hydrogel (2.00 ± 0.01%) and other commonly used biomaterials for wound healing [47]. These findings demonstrate that the hydrogel did not induce significant hemolytic reactions, suggesting its excellent erythrocyte compatibility.

### 2.4. Hemostatic Properties of Hydrogels

**Figure 5:**
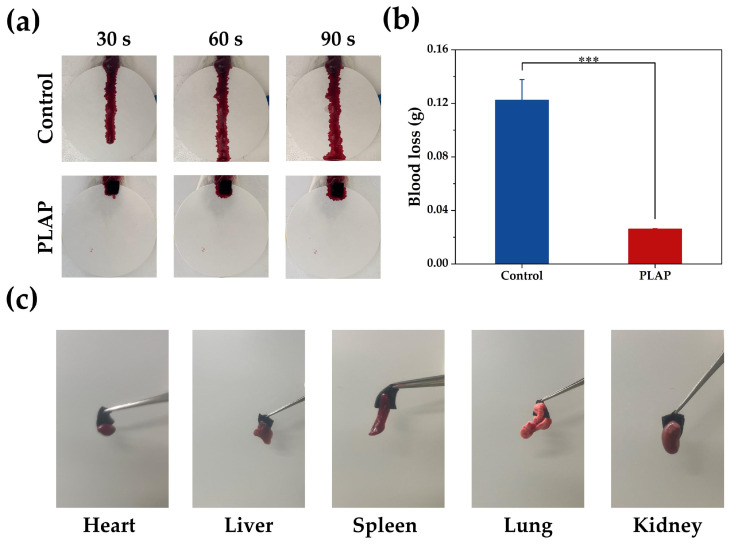
The hemostatic ability of hydrogels. (**a**) Photographs of the hemostatic effect in the liver of mice. (**b**) Liver blood loss in control group and hydrogel group (n = 3). (**c**) Tissue adhesion properties of PLAP hydrogels. A *p*-value of ≤ 0.05 was considered significant (*** *p* < 0.001).

Bleeding typically occurs during the initial stage of tissue damage. Timely hemostasis can reduce the inflammatory response and promote wound healing. The hemostatic performance of PLAP hydrogels was evaluated using a mouse liver hemorrhage model. PLAP hydrogels stopped liver bleeding in mice within 30 s (Figure 5a), and the liver blood loss (dry weight) was reduced to approximately 17% of that in the control group, demonstrating its excellent hemostatic activity (Figure 5b). When PLAP hydrogels come into contact with the wound, –OH and –NH on the surface of the hydrogels can form hydrogen bonds with the tissue to achieve tight adhesion to the tissue, thus effectively preventing blood overflow through physical sealing. As shown in Figure 5c, PLAP hydrogels rapidly adhere to various tissues, including heart, liver, spleen, lung and kidney. Moreover, when the hydrogels were lifted, the tissue remains attached, further confirming that PLAP hydrogels could effectively adhere to tissue and reduce wound bleeding. Furthermore, hydrogels have certain swelling properties, which can absorb blood exudates, thereby concentrating platelets and clotting factors, accelerating the clotting cascade and promoting the hemostatic process.

### 2.5. In Vitro Antibacterial Activity of Hydrogels

**Figure 6:**
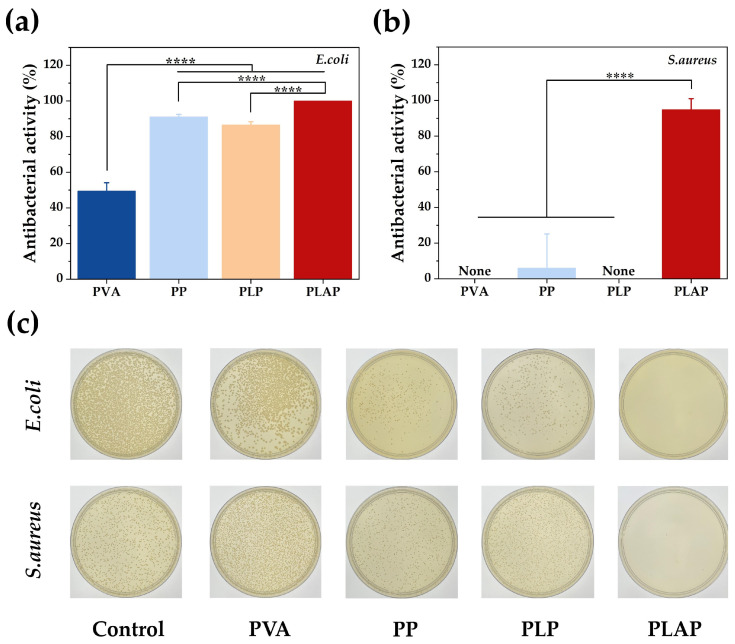
The antibacterial activity of hydrogels. (**a**) Antibacterial activity of hydrogels against *E. coli* and (**b**) *S. aureus.* (**c**) Colony plates of *E. coli* and *S. aureus* after co-culture with hydrogels. A *p*-value of ≤ 0.05 was considered significant (**** *p* < 0.0001).

Once the skin is damaged, bacterial infection can occur at any stage of wound healing, leading to an inflammatory response in the wound that impedes the healing process [48]. Therefore, qualified wound dressings should possess strong antibacterial properties. Escherichia coli (*E. coli*) and Staphylococcus aureus (*S. aureus*) are common strains responsible for wound infection. To evaluate the antibacterial activity of hydrogels, bacteria were co-cultured with the hydrogels. As shown in Figure 6a,c, the bactericidal rate of PVA against *E. coli* was 49.40 ± 4.77%. This antibacterial activity was attributed to the ability of PVA to inhibit the formation of *E. coli* biofilms [49]. Following the incorporation of PPy, the antibacterial activity of PP and PLP hydrogels against *E. coli* were significantly enhanced, with the bactericidal rates of 91.01 ± 1.46% and 86.55 ± 1.78%, respectively. This enhancement was primarily due to the presence of numerous of N-H activity groups in the PPy chain, which can interact electrostatically with the bacterial cell wall and bind bacterial cell proteins, leading to bacterial inactivation and death [50]. Upon further incorporation of Ag NPs, which function as a potent antimicrobial agent, the antibacterial activity of PLAP hydrogels was further improved, achieving a bactericidal rate close to 100%. However, in experiments conducted with *S. aureus*, PVA, PP and PLP hydrogels exhibited no significant antibacterial activity (Figure 6b,c), as previously reported [51]. In contrast, PLAP hydrogels demonstrated substantial antibacterial activity against *S. aureus*, with a bactericidal rate of 94.86 ± 6.18%. The reason for the above results is that the composition and structure of the cell walls of *E. coli* and *S. aureus* are different. *S. aureus* possesses a thick peptidoglycan layer, which is effective against antimicrobial agents. Unlike the double-membrane cell wall structure of *E. coli*, *S. aureus* is surrounded by a single membrane composed predominantly of lysine phosphatidylglycerol (Lys-PG). Lys-PG carries a positive charge, which makes *S. aureus* membranes resistant to the membrane-disrupting properties of cationic antibacterial materials [52]. Ag NPs are broad-spectrum antimicrobial agents with strong bactericidal capabilities. They exert their antibacterial effects through multiple mechanisms: interacting with negatively charged membrane phospholipids of bacteria to disrupt cell wall integrity; binding bacterial DNA to inhibit replication while disrupting cytochrome-mediated electron transport; and inducing oxidative stress to impair metabolic processes, ultimately leading to bacterial death [53,54]. Notably, Ag NPs can also effectively suppress biofilm formation, thereby preventing the development of bacterial resistance [55]. In addition, the small size and large specific surface area of Ag NPs facilitate their entry into bacterial interiors, enhancing their antimicrobial effects [56]. The results indicate that PLAP hydrogels are effective dressing for preventing bacterial infection and promoting wound healing.

### 2.6. In Vivo Wound Healing

**Figure 7:**
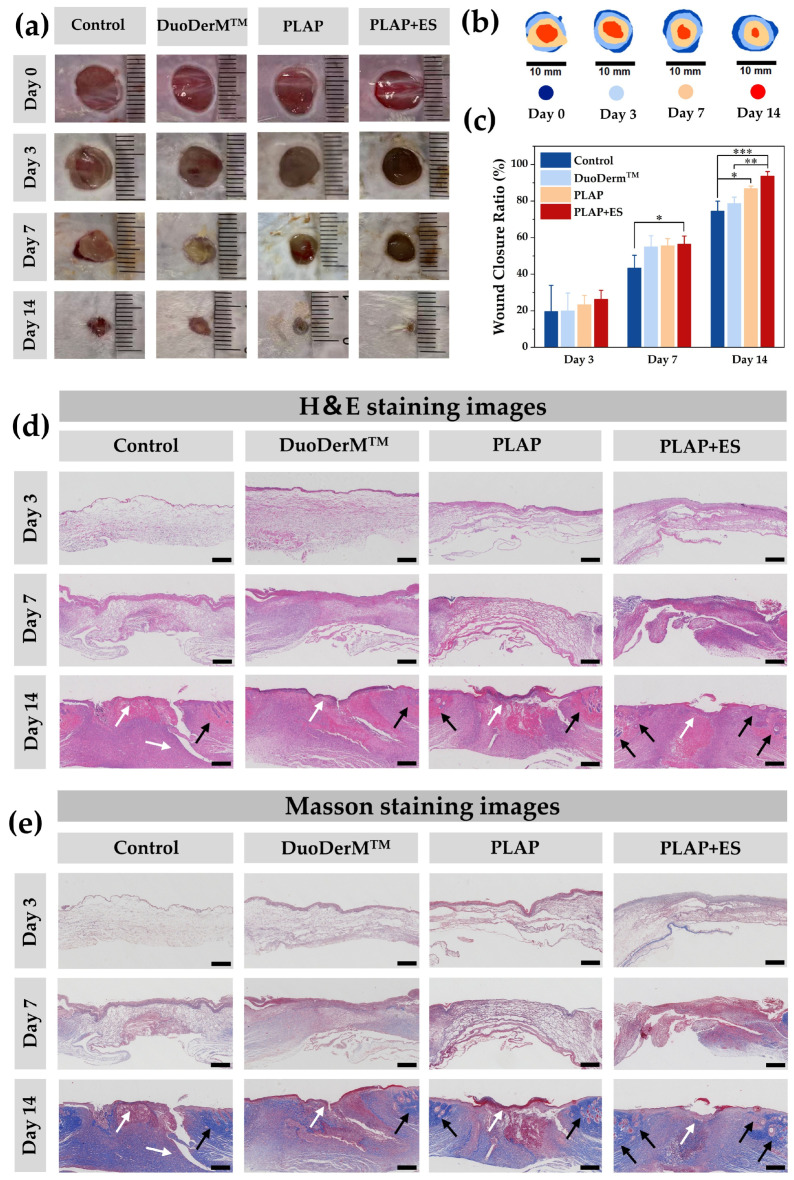
In vitro wound healing properties. (**a**) Representative photographs of mice with full-thickness wounds after treatment with different dressings on 3rd, 7th and 14th days. (**b**) Diagram of wound healing on 3rd, 7th and 14th days. (**c**) Wound closure rates on 3rd, 7th and 14th days after dressing treatment. (**d**) H&E and (**e**) Masson staining of wounds treated with different dressings on 3rd, 7th and 14th days. Wound tissues are indicated by white arrows and skin appendages are indicated by black arrows. (Scale bar = 500 μm)**.** A *p*-value of ≤ 0.05 was considered significant (* *p* < 0.05, ** *p* < 0.01, *** *p* < 0.001).

In vitro studies have demonstrated the potential of PLAP hydrogels as wound dressings for treating diabetic chronic wounds. Based on these findings, a full-thickness diabetic skin wound model was established to evaluate the ability of hydrogels to accelerate wound healing. Mice were injected with STZ, and full-thickness wounds of 8 mm in diameter were created in those with blood glucose levels exceeding 16.7 mmol/L for a week. Wounds were randomly treated with Tegaderm membrane (control group), DuoDerM^TM^ hydrogels, PLAP hydrogels and PLAP hydrogels + ES. Wound tissues were observed and collected on the 3rd, 7th and 14th days. As shown in Figure 7a, the PLAP hydrogel group and the PLAP+ ES group performed significantly better than other groups in promoting diabetic wound healing. Notably, the PLAP + ES group had the smallest wound area among the four groups at all time points, and the wound healing rate reached 93.71 ± 2.46% on the 14th day (Figure 7b,c).

To further evaluate the effects of the four dressing groups on regenerated wound tissue, H&E staining and Masson staining were used for histological analysis. Partial wound tissue in each group gradually regenerated on the 3rd, 7th and 14th days. As shown in Figure 7d, the results of H&E staining on the 14th day showed that the wound area of the control group was larger, the epidermis near the wound had not yet formed, and there were fewer skin appendages such as hair follicles, and the inflammatory reaction was more serious. In contrast, the DuoDerM^TM^ group showed reduced wound size, partial epidermal coverage and attenuated inflammation, though skin appendage regeneration remained limited. The PLAP hydrogel group demonstrated further improvements, including significantly smaller wounds, thicker epidermis, increased appendage density and mild inflammation. Notably, the PLAP + ES group achieved optimal outcomes: minimal wound area, robust epidermal thickening, abundant appendage regeneration and mild inflammation. In addition, Masson staining further confirmed superior healing in the PLAP + ES group, with more uniform collagen deposition and organization (Figure 7e). These results suggest that the PLAP + ES group has the best wound healing performance by accelerating re-epithelialization processes such as epidermal regeneration and skin accessory organ formation, as well as reducing wound inflammatory response and improving collagen deposition.

## 3. Conclusions

In summary, we have developed PLAP hydrogels dressing for the effective treatment of diabetic wounds. PLAP hydrogels were composed of PVA, Py, Lgs and Ag NPs, which were obtained by a one-pot method and cyclic freeze–thaw. The introduction of Lgs improved the water dispersion and electrical conductivity of PPy and served as a reducing agent for the in situ synthesis of Ag NPs. Through the combination of PPy and Lgs-Ag NPs, the conductivity of the hydrogel was further enhanced, reaching 2.82 ± 0.04 mS/cm, which was similar to that of normal skin tissue, meeting the needs of electrical stimulation treatment of chronic wounds. In vitro biocompatibility testing demonstrated that PLAP hydrogel had good biocompatibility, and the hemolysis rate was only 2.1 ± 0.38%, which suggested the hydrogel had good erythrocyte compatibility. In addition, the incorporation of Ag NPs makes the antibacterial rate of hydrogel against *E. coli* and *S. aureus* exceed 90%, effectively preventing wound infection. In vivo studies revealed that PLAP hydrogels can effectively promote wound healing. This treatment effect was further improved under the action of ES, the wound healing rate reached 93.71 ± 2.46% within 14 days, and the epithelial tissue formation was more complete, including collagen tissue and skin accessory organs. These results suggest that PLAP hydrogels are expected to be an effective platform for ES treatment of chronic wounds. In the follow-up study, the long-term stability of the application process of hydrogel needs to be further discussed, and more composition combinations and larger animal experiments should be explored to evaluate the potential of hydrogels in clinical applications.

## 4. Materials and Methods

### 4.1. Materials

Ammonium persulfate (APS) and streptozotocin (STZ) were purchased from Macklin Biochemical Co., Ltd., (Shanghai, China). PVA, Lgs and Py were obtained from Aladdin Biochemical Co., Ltd., (Shanghai, China). Silver nitrate (AgNO_3_) and ammonium hydroxide (NH_3_·H_2_O) were provided by Sinopharm Chemical Reagent Co., Ltd., (Shanghai, China). DMEM complete culture medium was purchased from Corning Co., Ltd., (Corning, New York, NY, USA). PBS, CCK-8 reagent and Calcein-AM staining reagents were obtained from Beyotime Biotechnology Co., Ltd., (Shanghai, China) Trypticase Soy Broth (TSB) liquid medium and tryptone Soy Agar (TSA) solid medium were provided by Thermo Fisher Scientific Co., Ltd., (Waltham, MA, USA). ICR mice (male, 28–32 g) were purchased from Charles River Co., Ltd., (Beijing, China). Other chemical reagents were of analytical grade.

### 4.2. Synthesis and Characterization of Lgs-Ag NPs

The Lgs-Ag NPs were synthesized based on the previous literature with some modifications [57]. First, Lgs was dissolved in ultrapure water at 2% (*w*/*v*), centrifuged and filtered through a 0.22 μm filter membrane to remove insoluble impurities. The filtrate was then freeze-dried for further use. Then, an aqueous Lgs solution (20 mg/mL) was prepared by dispersing Lgs (1 g) in ultrapure water (50 mL). Simultaneously, the AgNO_3_-dissolved solution was prepared by dissolving AgNO_3_ (0.63 g) in ultrapure water (40 mL), followed by mixing with NH_3_·H_2_O (5 mol/mL, 10 mL). Subsequently, the AgNO_3_-dissolved solution and the Lgs solution were mixed at 300 rpm for 12 h at room temperature to generate Lgs-Ag NPs. Finally, the solution was then frozen and freeze-dried to obtain dried Lgs-Ag NPs. The morphologic characteristics of Lgs-Ag NPs were characterized by transmission electron microscopy (TEM, FEI, Talos F200X, Waltham, MA, USA). The XRD patterns of Lgs-Ag NPs were characterized by X-ray diffraction (XRD, Bruker, D8 Advance, Karlsruhe, Germany). The optical properties of Lgs-Ag NPs were measured via a UV–Vis spectrophotometer (Agilent, Cary 300, Santa Clara, CA, USA). The chemical structure of Lgs-Ag NPs was analyzed using a Fourier-transform infrared spectrometer (FITR, IS 50, Waltham, MA, USA). More experimental details are provided in the Supporting Information.

### 4.3. Preparation of the Hydrogels

First, PVA was dissolved in ultrapure water at 20% (*w*/*v*). Then, a certain amount of Lgs-Ag NPs was ultrasonically dispersed in ultrapure water. Py, PVA and APS were then added successively, with stirring at 400 rpm for 30 min, 10 min and 120 min, respectively. This process needs to be carried out in a dark environment. Finally, the solution was poured into the mold, frozen at −20 °C for 2.5 h and thawed at room temperature for 0.5 h. This freeze–thaw cycle was repeated three times to fabricate the hydrogels (PLAP). Hydrogels prepared without Ag NPs were designated as PLP, while those prepared without both Lgs and Ag NPs were designated as PP. PVA hydrogels served as the control group. The compositions and dosages of the PVA-based hydrogels are listed in Table S1.

### 4.4. Rheological Properties of Hydrogels

A rheometer (TA, DHR-2, New Castle, DE, USA) was employed to test the rheological properties of the hydrogels. More experimental details can be found in the Supporting Information.

### 4.5. Electrical Property of Hydrogels

The pre-gel solution (0.7 mL) was injected into silicone molds (diameter: 20 mm, height: 2 mm) for shaping, and then the conductivity of each hydrogel was measured using a four-probe tester (STZ2258C; Suzhou Jingge Electronics Co., Ltd., Suzhou, China).

### 4.6. Morphology of the Hydrogels

The morphology of the hydrogels was obtained by scanning electron microscopy (SEM, Hitachi, regulus 8230, Tokyo, Japan) at an accelerating voltage of 10 kV. Before imaging, the freeze-dried hydrogels were sputter-coated with gold for 90 s using ion beam sputtering (IBS; Hitachi, MC1000, Tokyo, Japan).

### 4.7. Porosity of Hydrogels

The porosity of each hydrogel was measured using an absolute ethanol method. More experimental details are provided in the Supporting Information.

### 4.8. Adhesion Properties of Hydrogels

Quantitative characterization of the adhesive properties of the hydrogels was demonstrated by applying them to adhesive surfaces of various materials, including rubber, plastic, glass, wood, steel and skin. The qualitative characterization of the adhesive properties of the hydrogels was obtained by the lap shear experiment. More experimental details can be founded in the Supporting Information.

### 4.9. Moisturizing, Swelling and Degradation Properties of Hydrogels

Experimental details of moisturizing, swelling and degradation properties of the hydrogel are provided in the Supporting Information.

### 4.10. Cell Viability

The cell viability was evaluated using an indirect contact method. After ultraviolet sterilization for 2 h, the hydrogels were mixed with DMEM complete culture medium at a ratio of 1:10, incubated in 37 °C incubators for 24 h and then filtered through a 0.22 μm filter membrane to prepare the extract for subsequent experiments.

L929 cells were seeded into 96-well plates (1 × 10^5^ cells/well) and cultured with 100 μL extract for 24 h, whereas the control group was cultured in 100 μL of DMEM complete medium. Six parallels were set in each group. After co-culture for 24 h, 48 h and 72 h, the medium was sucked off, 100 μL of PBS was added to gently wash off the remaining medium. Then, 100 μL of 10% CCK-8 solutionwas added, and the background hole was set to contain only CCK-8 solution. After incubation in the incubator for 1 h, the absorbance at 450 nm was measured using an enzyme labeler. Absorbance is proportional to cell viability. Calcein-AM staining reagentswere used for live/dead staining. Removing all the culture medium from the 96-well plates, 100 μL of PBS was then added to gently wash the remaining culture medium. After that, Calcein-AM staining solution was added, and the plates were incubated for 20 min at 37 °C. The cell morphology was observed under a fluorescence microscopy

(1) $$\mathrm{Cell}\;\mathrm{viability}\;\left(\%\right)=(A_{s}-A_{b})/(A_{c}-A_{b})\times 100$$

where A_s_, A_c_ and A_b_ represent the absorbance of the hydrogel groups, the control group and the CCK-8-only blank group, respectively.

### 4.11. In Vitro Hemolysis Assay

The hemolysis performance of each hydrogel was evaluated by the hemolysis ratio. Experimental details are provided in the Supporting Information.

### 4.12. Hemostatic Ability Evaluation

A mouse liver bleeding model was used to evaluate the hemostatic ability of the hydrogels in vitro [58]. More experimental details are provided in the Supporting Information.

### 4.13. Antibacterial Activity of Hydrogels

*E. coli* (ATCC 25922) and *S. aureus* (ATCC 6835) were selected as the experimental strains to evaluate the antibacterial activity of the hydrogels via a co-culture method. First, 10 μL of bacterial solution was added to 10 mL of TSB and cultured in an incubator at 37 °C overnight until the bacteria reached the logarithmic growth phase (OD: 0.6–0.8). The bacterial suspension was gradiently diluted 100 times by normal saline. Subsequently, 0.7 mL of bacterial suspension was added to 7 mL of normal saline containing 10% hydrogels for co-culture, and the bacteria treated without hydrogels were used as the control group. The cultures were then incubated in a shaking incubator at 37 °C for 4 h. Finally, 100 μL of bacterial suspension was inoculated onto TSA, evenly spread and incubated in 37 °C incubators for 18 h. The colonies were then counted. Each group was set up with three parallel samples. The antibacterial activity of PVA-based hydrogels can be calculated as follows:

(2) $$\mathrm{Antibacterial}\;\mathrm{activity}\;(\%)=(A_{p}-A_{c})/A_{c}\times 100\%$$

where A_p_ and A_c_ represent the numbers of bacterial colonies in the hydrogel and control groups, respectively.

### 4.14. In Vivo Wound Healing Assay

A type 1 diabetes model was established in male ICR mice weighing 28–32 g by intraperitoneal injection of prepared streptozotocin solution (dissolved in pH 4.3 citrate buffer). The first three doses were 50 mg/kg, with subsequent doses of 150 mg/kg until the blood glucose concentration of mice exceeded 16.7 mmol/L, at which point the model was considered to be successfully established. All mice were maintained in an SPF laboratory, housed five per cage with normal air circulation and food supply. The mice were anesthetized via intraperitoneal injection of sodium thiopental at a dose of 50 mg/kg. The dorsal fur of the mice was removed, the area was disinfected with alcohol, and a round full-thickness skin wound with a diameter of 8 mm was created on the skin with a hole punch. Mice were randomly allocated into four experimental groups (n ≥ 15 per group). For the control group, wounds remained untreated. In the commercial group, wounds were treated with DuoDerM^TM^ gel (Convatec, Berkshire, UK). In the PLAP group, the wounds were covered with the PLAP hydrogels. For the PLAP + ES group, wounds were treated with PLAP hydrogels combined with ES. The ES protocol was administered by attaching two wires to the hydrogel and the other end of the wires was connected to a 1.5V DC power supply. Each ES treatment session lasted 20 min and was repeated every two days, totaling three sessions. To prevent scratching, all wounds were covered with commercial Tegaderm™ 1624 W transparent dressing (3M, Saint Paul, MN, USA). At 3, 7 and 14 days, photographs of the wounds were captured using a mobile phone, and the wound areas were measured via image J software (1.54 f). Meanwhile, the mice were sacrificed, and then the newly regenerated skin was immediately harvested and fixed in 4% paraformaldehyde. Skin tissue sections were processed with H&E and Masson staining to assess pathological changes in regenerated tissue and collagen deposition, respectively. All animal experiments were approved by the Guoke Ningbo Life Science and Health Industry Research Institute in accordance with GB/T35892-2018 [59] with decision number GK-2024-XM-1048.

### 4.15. Statistical Analysis

All data in this study were analyzed using GraphPad Prism software (version 9.5.1) and Origin Pro (version 2024b). Each experiment was repeated at least three times (n = 3), and the results are presented as mean ± standard deviation (SD). The statistical significance of the two groups was analyzed using the paired Student’s *t*-test. For more groups, the statistical significance of the groups was analyzed using one-way analysis of variance (ANOVA). A *p*-value of ≤ 0.05 was considered significant (* *p* < 0.05, ** *p* < 0.01, *** *p* < 0.001, **** *p* < 0.0001).

## Data Availability

Data will be made available on request.

## Supplementary Materials

The following supporting information can be downloaded at: https://www.mdpi.com/article/10.3390/gels11040283/s1, Figure S1: Oscillatory strain scanning of PVA hydrogels with different concentrations; Figure S2: Cartogram for the particle size distribution of Lgs-Ag NPs; Figure S3: Microscopic characteristics of hydrogels. (a) SEM images of hydrogels (Scale bar = 10 μm). (b) The porosity of hydrogels; Figure S4: Adhesion properties of hydrogels; Figure S5: SEM images of (i) Lgs, (ii) PPy, (iii) Lgs@ PPy (Scale bar = 5 μm); Figure S6: Series circuit diagram of PLAP hydrogel and light emitting diode (LED); Table S1: Composition and Dosage of PVA-based hydrogels.
