# Supplementary material for: Lignin-Mediated Dual Conductive Hydrogels with High Conductivity, Antibacterial Activity and Biocompatibility for Chronic Wound Repair
Source: Gels. 2025 Apr 11;11(4):283. doi: 10.3390/gels11040283 (PMC12026575; doi:10.3390/gels11040283)
Supplement: Supplementary file 1 [file gels-11-00283-s001.zip › gels-3553300-supplementary.pdf]

# Supporting Information

# Lignin-mediated dual conductive hydrogels with high conductivity, antibacterial activity and biocompatibility for chronic wound repair

**Jianhong Lin [1,2,†], Mengyao Chen [1,3,†], Wei Zhao [1,4], Shengyu Zhang [1,4], Jialin Liu [1,2], Yang Zhou [1,2], Lei Jiang [1,*], Jiantao Zhang [1,2,*]**

[1] Laboratory of Advanced Theranostic Materials and Technology, Ningbo Institute of Materials Technology and Engineering, Chinese Academy of Sciences, Ningbo 315201, China

[2] University of Chinese Academy of Sciences, Beijing 100049, China

[3] School of Materials Science and Chemical Engineering, Ningbo University, Ningbo, 315201, China

[4] Cixi Biomedical Research Institute, Wenzhou Medical University, Cixi 315302, China

[†] These authors contributed equally to this work.

* Correspondence: jianglei@nimte.ac.cn (L.J.); zhangjiantao@nimte.ac.cn (J.Z.)

## 1. Characterization of Lgs-Ag NPs

The microstructure of Lgs-AgNPs was analyzed by depositing 10 μL droplets of a 0.05 mg/mL Lgs-AgNPs solution onto a copper grid, drying at room temperature until complete solvent evaporation, and subsequently observing under an electron microscope (TEM, FEI, Talos F200X, Waltham, Massachusetts, USA).

The XRD pattern of Lgs-AgNPs was acquired using an X-ray diffraction (XRD, Bruker, D8 Advance, Karlsruhe, Germany), with the powder sample loaded on a zero-background holder, scanned over a 2θ range of 10° to 80°.

The UV-Vis absorption spectrum of Lgs-AgNPs was measured using a UV–vis spectrophotometer (Agilent, Cary 300, Santa Clara, California, USA) after preparing an aqueous solution (0.05 mg/mL). The scanning wavelength range was set from 200 to 800 nm.

The FTIR spectra of Lgs-Ag NPs were acquired using a Fourier-transform infrared spectrometer (FITR, IS 50, Waltham, Massachusetts, USA). The Lgs-Ag NPs was analyzed in dry form using ATR mode with a wavenumber range of 4000-400 $cm^{-1}$.

## 2. Rheological of hydrogels

A rheometer (TA, DHR-2, New Castle, Delaware, USA) was employed to test the rheological properties of hydrogels. The tests were carried out on a 20 mm parallel plate. The linear viscoelastic region of the hydrogels were determined through oscillatory strain sweeps, with strain amplitude ranging from 0.01 to 10000%, a shear frequency of 10 rad/s, and a temperature of 25℃. The dynamic viscoelasticity of hydrogels were determined through oscillatory frequency sweep measurements. The tests were conducted at an angular frequency 0.1 ~ 100 rad/s, a shear strain 1.0%, and a temperature of 25℃.

## 3. Porosity of hydrogels

The porosity of hydrogels was measured by absolute ethanol method. First, the freeze-dried hydrogel was cut into a specification of 10 mm×10 mm×0.02 mm, the volume was recorded as v, and the weight was recorded as $A_c$. Then the hydrogels were immersed in anhydrous ethanol and kept in a vacuum environment for 10 minutes to ensure that no bubbles were formed. The hydrogels were removed from absolute ethanol, and the surface ethanol of hydrogels were gently blotted dry, the weight of hydrogels was recored as $A_s$. The porosity of hydrogel was calculated by the following formula:

$$\text{Hemolysis ratio (\%)} = (A_s-A_c)/\varrho)/v\times100 \quad (1)$$

where $A_s$, $A_c$, ϱ and v represent the initial weight of the hydrogel, the weight of the hydrogel after absorption of absolute ethanol, the density of absolute ethanol, and the volume of the hydrogel, respectively.

## 4. Adhesion properties of hydrogels

The adhesion properties of hydrogels were qualitatively characterized by pressing them onto surfaces of various materials (e.g., rubber, plastic, glass, wood, steel, and skin) and then recording the adhesion of the hydrogel. Quantitative analysis was performed via a lap shear test. Hydrogel samples were prepared as round sheets (20 mm in diameter, 2 mm thick) and bonded to porcine skin, followed by adhesive strength measurement using

a universal testing machine (UTM, CMT 5205, Eden Prairie, Minnesota, USA).

## 5. Moisturizing, swelling and degradation properties of hydrogels

700 mg of hydrogel were placed in an incubator (37℃, 75% RH). Weight measurements were taken at predetermined time intervals, and the water loss ratio was calculated using the following formula:

$$\text{Water loss ratio (\%)}= (A_c\text{-}A_t)/A_c\times 100 \quad (2)$$

where $A_c$ and $A_t$ represent the initial weight and the weight at a fixed point in time of the hydrogel, respectively.

500 mg of hydrogel were immersed in 5 mL of PBS (pH 7.2) and incubated in an incubator at 37 °C. At predetermined time intervals, the hydrogel samples were taken out, surface moisture was gently blotted dry, and mass changes were recorded. The swelling ratio was then calculated using the following equation:

$$\text{Swelling ratio (\%)}= (A_t\text{-}A_c)/A_c\times 100 \quad (3)$$

where $A_c$ and $A_t$ represent the initial weight and the weight at a fixed point in time of the hydrogel, respectively.

500 mg of hydrogel were immersed in 5 mL of PBS (pH 7.2) and incubated in an incubator at 37 °C. At predetermined time intervals, the hydrogel samples were removed from the PBS solution, rinsed with deionized water to remove surface residues, and gently blotted with filter paper to eliminate excess moisture, then, the mass change of the hydrogel was measured. The degradation ratio was calculated using the following equation:

$$\text{Degradation ratio (\%)} = (A_c\text{-}A_t)/A_c\times 100 \quad (4)$$

where $A_c$ and $A_t$ represent the initial weight and the weight at a fixed point in time of the hydrogel, respectively.

## 6. In Vitro Hemolysis Assay

Fresh rabbit whole blood was collected and mixed in an anticoagulant tube containing EDTA. The rabbit blood was transferred into a centrifuge tube, centrifuged at 300 ×g for 10 min, and the precipitated blood cells at the bottom were collected. Blood cells (1 mL) were mixed with 10 mL PBS, centrifuged at 1000 ×g for 10min, repeat two to three times until the supernatant became colorless and transparent. The blood cells were then diluted with saline to prepare a 5% (v/v) suspension for later use. Hydrogels (0.2 mL) were added to a 5 mL centrifuge tube, mixed with 1.8 mL blood cell suspension, and incubated on a shaking table at 37°C for 1 h. Negative and positive controls were replaced with 0.2 mL saline and 0.2 mL 1% TritonX-100, respectively. Then the reacted blood was thoroughly mixed and transferred into a 2 mL centrifuge tube, centrifuged at 1000 ×g for 10 min, and the samples were placed on the same level. The hemolysis phenomenon was photographed by a mobile phone. The supernatant of 100 μL sample was sucked into the 96-well plate and the absorbance of the sample at 540 nm was measured. Each group conducted three parallel experiments.

$$\text{Hemolysis ratio (\%)} = (A_s - A_b)/(A_c - A_b) \times 100 \tag{5}$$

where $A_s$, $A_c$, and $A_b$ represent the absorbance of the hydrogel, the 1 % TritonX-100, and saline groups, respectively.

## 7. Hemostatic Ability Evaluation

Four-week-old male ICR mice were anesthetized via intraperitoneal injection of sodium thiopental (50 mg/kg). The mice were placed in a supine position, and an incision was made near the sternum to expose the liver. Surface tissue fluid and blood were gently wiped away with a cotton swab. A pre-weighed filter paper was placed under the liver. A 3 mm incision was then made on the liver surface using a scalpel, and the incision site was immediately covered with 200 μL hydrogels. The control group received no treatment. The blood loss from the incision within 90 s was determined by measuring the weight change of the filter paper. Meanwhile, the bleeding process was recorded by phone.

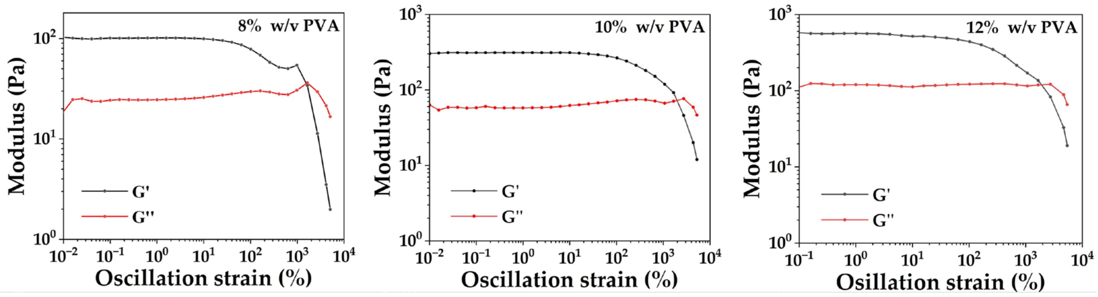


**Figure S1.** Oscillatory strain scanning of PVA hydrogels with different concentrations.

**Table S1.** Composition and Dosage of PVA-based hydrogels

| Sample | PVA (20%w/v) | Py | Lgs | Lgs-Ag NPs | APS (0.029M) | ultrapure |
|---|---|---|---|---|---|---|
| PVA | 3.0 ml | 0 | 0 | 0 | 0 | 2.0 ml |
| PP | 3.0 ml | 0.05 g | 0 | 0 | 0.75 ml | 1.25 ml |
| PLP | 3.0 ml | 0.025 g | 0.025 g | 0 | 0.75 ml | 1.25 ml |
| PLAP | 3.0 ml | 0.025 g | 0 | 0.025 g | 0.75 ml | 1.25 ml |

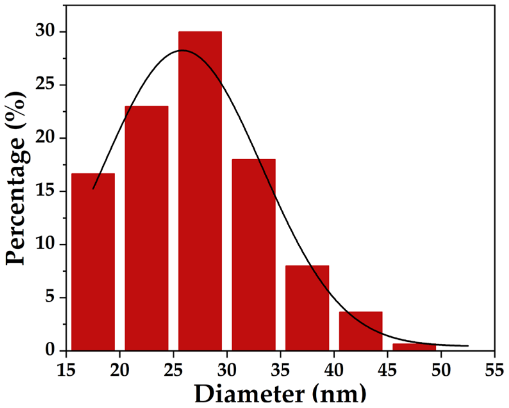


**Figure S2.** Cartogram for the particle size distribution of Lgs-Ag NPs.

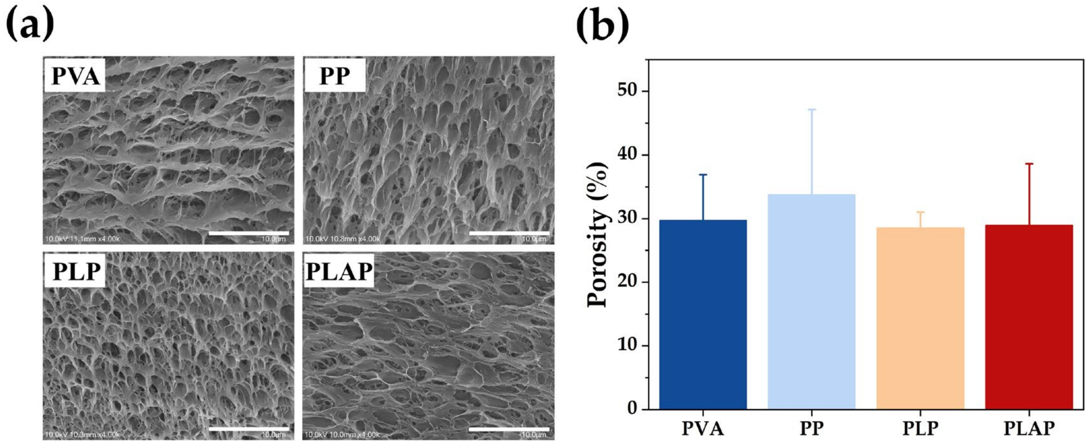


**Figure S3.** Microscopic characteristics of hydrogels. (a) SEM images of hydrogels (Scale bar = 10 μm). (b) The porosity of hydrogels.

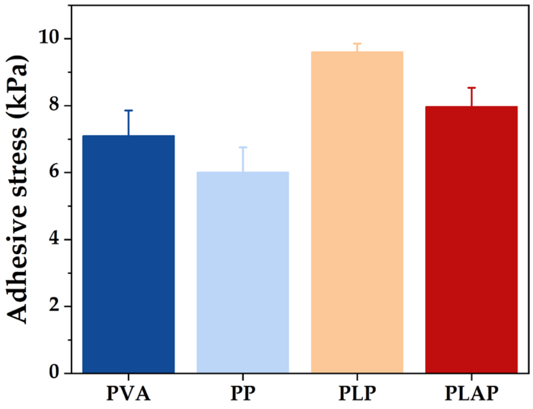


**Figure S4.** Adhesion properties of hydrogels.

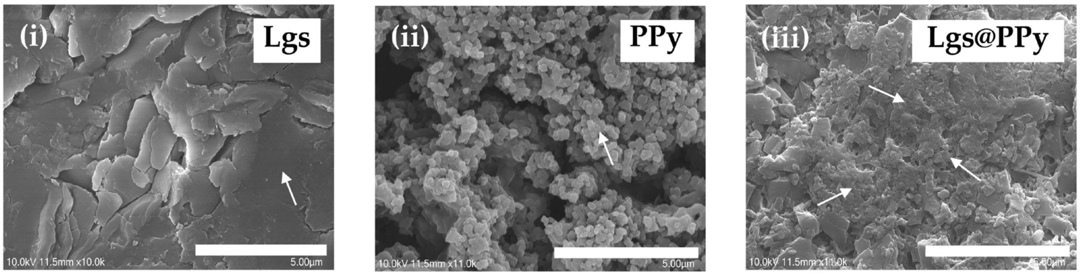


**Figure S5.** SEM images of (i) Lgs, (ii) PPy, (iii) Lgs@ PPy (Scale bar = 5 μm).

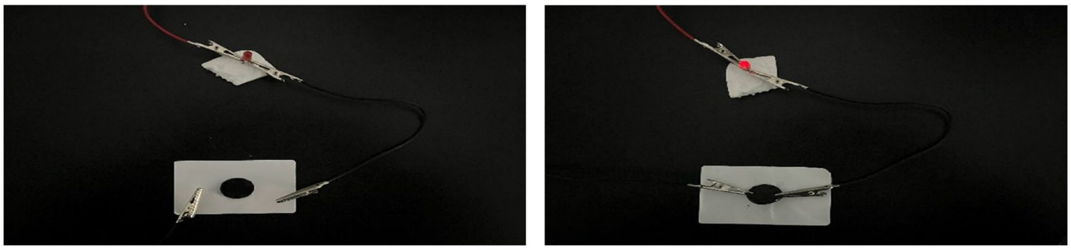

**Figure S6.** Series circuit diagram of PLAP hydrogel and light emitting diode (LED).
